# *TOMOMAN*: a software package for large-scale cryo-electron tomography data preprocessing, community data sharing and collaborative computing

**DOI:** 10.1107/S1600576724010264

**Published:** 2024-11-22

**Authors:** Sagar Khavnekar, Philipp S. Erdmann, William Wan

**Affiliations:** a Max Planck Institute of Biochemistry Martinsried Germany; b Human Technopole, Milan, Italy; c Department of Biochemistry, Center for Structural Biology Vanderbilt University School of Medicine Nashville TN37232 USA; Oak Ridge National Laboratory, USA; North Carolina State University, USA

**Keywords:** *TOMOMAN*, cryo-electron tomography, cryo-ET, structural biology, cryo-electron microscopy, cryo-EM, subtomogram averaging

## Abstract

Cryo-electron tomography (cryo-ET) and subtomogram averaging (STA) are becoming the preferred methodologies for investigating subcellular and macromolecular structures in native or near-native environments. Although cryo-ET is amenable to a wide range of biological problems, these problems often have data-processing requirements that need to be individually optimized, precluding the notion of a one-size-fits-all processing pipeline. Cryo-ET data processing is also becoming progressively more complex due to the increasing number of packages for each processing step. Though each package has its strengths and weaknesses, independent development and different data formats make them difficult to interface with one another. *TOMOMAN* (*TOMOgram MANager*) is an extensible package for streamlining the interoperability of packages, enabling users to develop project-specific processing workflows. *TOMOMAN* does this by maintaining an internal metadata format and wrapping external packages to manage and perform preprocessing, from raw tilt-series data to reconstructed tomograms. *TOMOMAN* can also export these metadata between various STA packages. *TOMOMAN* includes tools for archiving projects to data repositories, allowing subsequent users to download *TOMOMAN* projects and directly resume processing. By tracking essential metadata, *TOMOMAN* streamlines data sharing, which improves the reproducibility of published results, reduces computational costs by minimizing reprocessing, and enables the distribution of cryo-ET projects between multiple groups and institutions. *TOMOMAN* provides a way for users to test different software packages in order to develop processing workflows that meet the specific needs of their biological questions and to distribute their results to the broader scientific community.

## Abstract 2

Here we describe *TOMOMAN* (*TOMOgram MANager*), an extensible open-source software package for handling cryo-electron tomography data preprocessing. *TOMOMAN* streamlines interoperability between a wide range of external packages and provides tools for project sharing and archiving.

## 1. Introduction

Cryo-electron tomography (cryo-ET) is emerging as the method of choice for determining the structures of biological macromolecules *in situ*, that is, within their native context inside cells or intact extracellular particles (Young & Villa, 2023 ▸; McCafferty *et al.*, 2024 ▸). Unlike other cryo-electron microscopy (cryo-EM) approaches such as single particle analysis (SPA), cryo-ET provides direct 3D reconstructions of specific fields of view, allowing one to resolve individual molecules in thick complex specimens that would otherwise be overlapping in 2D projections. In cryo-ET, the sample is physically tilted in the microscope and projections are acquired at discreet tilt angles, resulting in a tilt-series that contains multiple distinct views of the same region of interest; these tilt-series can then be used to reconstruct 3D tomographic volumes. Subsequent high-resolution structure determination can be performed using subtomogram averaging (STA), an approach analogous to SPA methods in cryo-EM, where repeating units in the tomographic data can be extracted, aligned, averaged and classified (Wan & Briggs, 2016 ▸; Pyle & Zanetti, 2021 ▸; Castaño-Díez & Zanetti, 2019 ▸).

**Figure 1:**
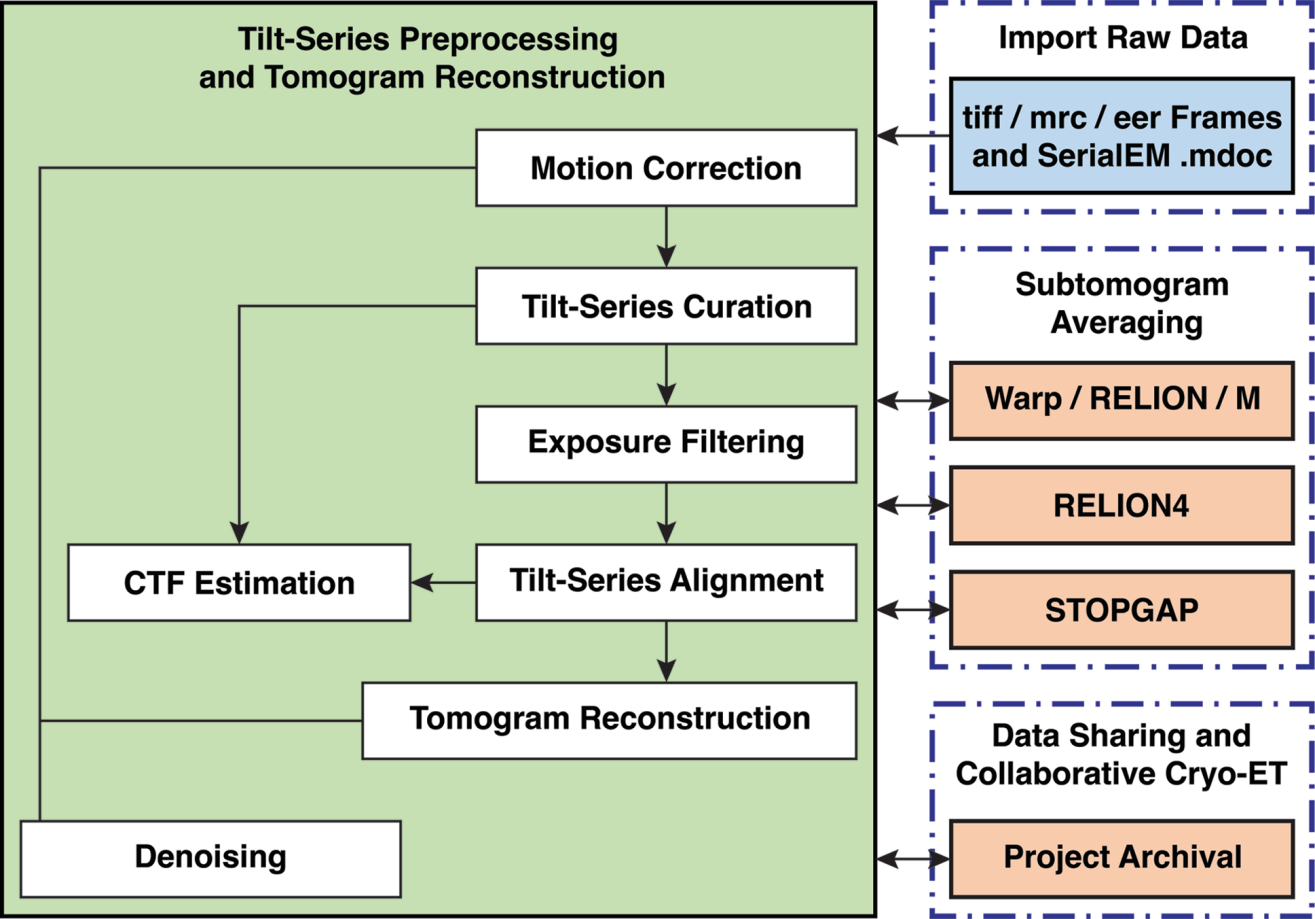
Example *TOMOMAN* workflow. *TOMOMAN* imports raw tilt-series frames and corresponding .mdoc files (top right). Preprocessing is highlighted in the light-green box and individual *TOMOMAN* modules are depicted as white boxes; data flow is indicated by the arrows. Preprocessed tilt-series and reconstructed tomograms can then be exported to subsequent STA workflows (center right), including *STOPGAP*, *Warp/RELION/M* and *RELION4*. Any given state of the *TOMOMAN* project can be archived and can facilitate collaborative cryo-ET (bottom right).

Going from tilt-series data acquisition to reconstructing a tomogram involves a large number of image-processing tasks (Fig. 1 ▸). We refer to this part of the cryo-ET processing workflow as ‘preprocessing’, which occurs prior to downstream analysis such as STA. Preprocessing starts with motion correction (Zheng *et al.*, 2017 ▸; Brilot *et al.*, 2012 ▸) followed by the assembly of the tilt-series in an ascending order of acquisition tilt angles (Hagen *et al.*, 2017 ▸). Manual curation of this tilt-series is then performed to remove bad images such as those with poor tracking, drift or specimen charging. Curated tilt-series are then exposure filtered to reduce the high-resolution noise in later images (Schur *et al.*, 2016 ▸; Grant & Grigorieff, 2015 ▸). These exposure-filtered tilt-series must be aligned using either fiducial-based (Mastronarde & Held, 2017 ▸) or fiducial-less approaches (Zheng *et al.*, 2022 ▸; Mastronarde & Held, 2017 ▸). Prior to tomogram reconstruction, the contrast transfer function (CTF) needs to be estimated; this can occur directly after tilt-series curation (Rohou & Grigorieff, 2015 ▸) or after tilt-series alignment to make use of the alignment information (Khavnekar & Wan, 2024 ▸). Various tomographic reconstructions can then be generated, such as raw (Kremer *et al.*, 1996 ▸), CTF-corrected (Turoňová *et al.*, 2017 ▸; Xiong *et al.*, 2009 ▸) or denoised tomograms (Buchholz *et al.*, 2019 ▸). The choice of which type of tomogram to reconstruct is determined by the use case; CTF-corrected tomograms provide high-resolution information for STA while denoised tomograms provide improved contrast, which can be useful for direct visual analysis.

Accurate preprocessing determines the success of all downstream analysis (Voortman *et al.*, 2014 ▸), spurring an increasing number of new approaches for each of these preprocessing tasks. These packages are often developed by different groups with different design philosophies, and with different inputs, outputs and file formats (Watson & Bartesaghi, 2024 ▸). As such, combining various packages to tailor workflows for specific biological problems is often complex and requires significant user effort.

*TOMOMAN* (*TOMOgram MANager*) is an extensible MATLAB-based package developed to reduce the complexity of combining different packages in order to streamline the testing and development of cryo-ET preprocessing and STA workflows. *TOMOMAN* achieves this by maintaining the essential metadata in an HDF database file (MATLAB structure format) for each tilt-series in the dataset. These metadata include information such as file names and paths, data collection parameters, preprocessing parameters such as estimated defocus, and tilt-series alignment information. *TOMOMAN* mainly acts as a wrapper for external packages, storing relevant metadata in its internal format and managing the input, output and running of external packages. This includes managing resources for parallel processing in high-performance computing environments. As part of its design ethos, *TOMOMAN* is meant to be pipeline agnostic, where we make no assumption of an ideal or standard processing workflow. *TOMOMAN* is designed to help interface the various software packages used in each step or task of cryo-ET preprocessing so that users can test and determine the best workflow for their specific biological projects.

For subsequent STA, various pieces of preprocessing metadata are required, though the specific types and formatting of these metadata are different for each STA package. As with preprocessing, *TOMOMAN* facilitates the export of tilt-series data and preprocessing metadata in the correct format for each STA package, such as *STOPGAP* (Wan *et al.*, 2024 ▸), *RELION* (Zivanov *et al.*, 2022 ▸; Bharat & Scheres, 2016 ▸) and *Warp/M* (Tegunov & Cramer, 2019 ▸; Tegunov *et al.*, 2021 ▸). Furthermore, *TOMOMAN* can also facilitate the transfer of projects between these STA packages to enable users to make use of their specific strengths.

As the cryo-ET field expands and an increasing amount of experimental data are collected, it is becoming virtually impossible for a single group to possess the requisite human effort, computational resources and biological expertise to fully analyze all the information within the data-rich tomograms. As such, there is a need for simultaneous collaborative efforts to fully leverage these data to yield novel biological insights; these efforts can take the form of inter-institutional collaborations or larger research consortia. However, such collaborative efforts are still unlikely be able to completely analyze all the biological information, making open data archiving a requirement. Current practices of depositing either raw frame data, partially processed data such as tilt-series or final reconstructed tomograms are not sufficient as they do not include all the necessary information to reproduce published results or provide consistency to others that perform additional preprocessing. Furthermore, repeating preprocessing tasks using the same methods becomes a significant waste of resources. Maintaining consistency between independent preprocessing efforts is paramount for building richly annotated, accessible data that provide biological insights to the broader community beyond structural biologists. Beyond streamlining cryo-ET workflows, *TOMOMAN* also enables reproducible data sharing and archiving. One example of such a distributed project is the recently released EMPIAR-11830 dataset (Khavnekar *et al.*, 2023*a* ▸; Waltz *et al.*, 2024 ▸), which has been deposited as a *TOMOMAN* minimal project (see below). The well defined metadata structure of *TOMOMAN* has already proven useful as the CZII visualization portal was able to intake these data for further computation (https://cryoetdataportal.czscience.com/datasets/10302), having run automated membrane segmentation on the whole dataset (Lamm *et al.*, 2024 ▸).

## 2. The *TOMOMAN* workflow

### 2.1. Overview of *TOMOMAN*

*TOMOMAN* is an open-source package written in MATLAB that can be run in three different ways. The first is as a MATLAB toolbox that can be sourced and directly used in MATLAB with a license. The second is a pre-compiled standalone package, which allows for interactive command-line use via the freely available MATLAB Compiler Runtime, similar to the *Dynamo* subtomogram averaging package (Castaño-Díez *et al.*, 2012 ▸; Castaño-Díez, 2017 ▸). The third is a pre-compiled ‘parallel’ executable, which can be used in high-performance parallel computing environments using workload managers such as *SLURM* (Yoo *et al.*, 2003 ▸). In addition to managing metadata, the *TOMOMAN* parallel executable also transparently manages the computational resource usage of external packages.

**Figure 2:**
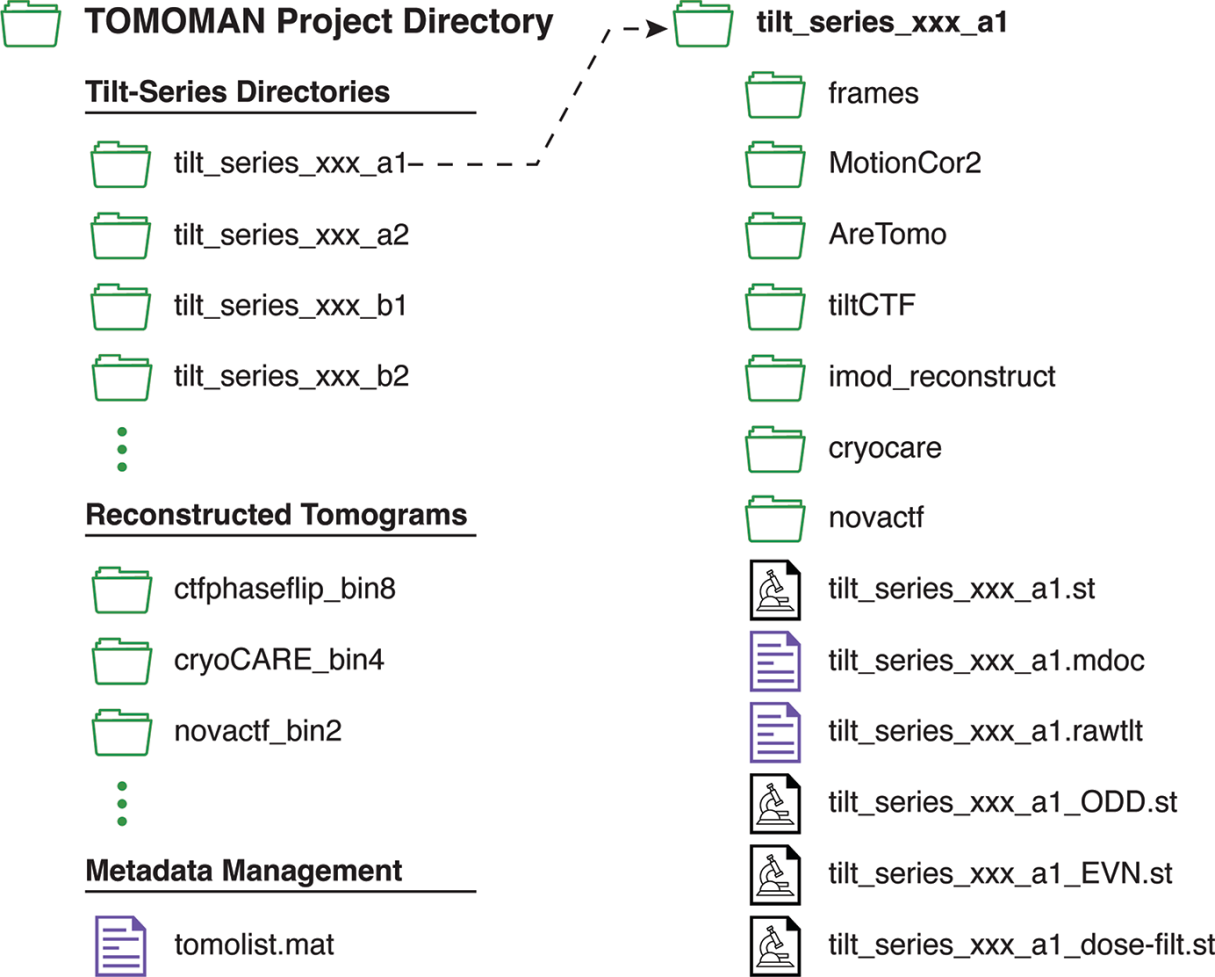
Example *TOMOMAN* project directory structure. The main project directory includes subfolders for each tilt-series, reconstructed tomograms and metadata files such as the tomolist. Each tilt-series subdirectory includes further subdirectories for raw tilt-series frames and individual preprocessing tasks. Tilt-series directories also contain original microscope metadata in *SerialEM* format, motion-corrected and curated tilt-series, corresponding odd and even frame tilt-series, and dose-filtered stacks if performed.

At the core of *TOMOMAN* is the internal metadata format and a well defined directory structure (Fig. 2 ▸). Within the root directory of each project, a subdirectory is generated for each tilt-series in the dataset; all subsequent preprocessing tasks are performed within these subdirectories. Each tilt-series directory contains a subdirectory with the raw frames, the raw non-motion-corrected tilt stack (if available) and the corresponding *SerialEM*-formatted tilt-series acquisition metadata (*i.e.* the .mdoc file) (Mastronarde, 2005 ▸). For each preprocessing step, the intermediate input and output data are stored in a subdirectory named for the software package used. The relevant metadata generated by each preprocessing step are parsed and stored in an HDF-formatted file in the project root directory; this file is referred to as the ‘tomolist’. Reconstructed tomograms are not considered essential data, as they are often very large and can be readily reconstructed from the annotated metadata faster than they can be transmitted. As such, tomograms are stored in their own directories separate from the tilt-series directories.

Below are descriptions of each preprocessing task performed in *TOMOMAN*. Each of these tasks is configured using package-specific *TOMOMAN* parameter files, which are a set of plain-text files that include the parameters for each preprocessing step as name–value pairs. The parameters generally include several *TOMOMAN*-specific parameters and, for external packages, each input parameter for that package. *TOMOMAN* tasks are programmed as modules within the package, allowing for the addition of new software packages to accomplish pre-existing tasks or to develop new preprocessing tasks. Currently supported packages will be described in the documentation of each *TOMOMAN* release.

### 2.2. Importing microscope data

The first step in *TOMOMAN* preprocessing is importing raw microscope data and sorting them into subdirectories for each tilt-series. During this step, *TOMOMAN* scans a designated raw data directory for .mdoc files. For each one it finds, it generates a tilt-series directory. For new projects, *TOMOMAN* also initializes the tomolist; repeated importing will append the previous tomolist with new data, allowing for continuous *TOMOMAN* preprocessing during data acquisition.

### 2.3. Generating motion-corrected tilt-series

After raw microscope data have been imported, frames need to be motion-corrected and subsequent aligned images assembled into a tilt-series. *TOMOMAN* includes modules for performing motion correction using either *MotionCor2* (Zheng *et al.*, 2017 ▸) or using *RELION*’s implementation of *MotionCor2* (Zivanov *et al.*, 2019 ▸). After motion correction, *TOMOMAN* assembles the summed frame-aligned images into tilt-series by tilt angle, accounting for the tilt acquisition scheme used (Hagen *et al.*, 2017 ▸). *TOMOMAN* can also output odd and even frame stacks for later use in *noise2noise*-based denoising (Buchholz *et al.*, 2019 ▸).

### 2.4. Curating and cleaning tilt-series data

After assembly of motion-corrected tilt-series, manual curation of tilt images is often necessary to remove ‘bad’ images. Bad images include those where something blocks the field of view, such as a grid bar or ice crystal, or image quality is poor due to issues such as sample charging or drift. *TOMOMAN* facilitates this curation process using the *clean_stacks* task, which opens each tilt-series in *IMOD’*s *3dmod* program (Kremer *et al.*, 1996 ▸), requests input on bad images and removes the bad images from tilt-series while annotating this in the tomolist. Tilt-series that are completely bad can also be noted in the tomolist, so that they can be removed from further preprocessing tasks.

### 2.5. Exposure filtering

Exposure filtering significantly improves the contrast, alignment quality and high-resolution signals of tilt-series and reconstructed tomograms (Schur *et al.*, 2016 ▸; Wan & Briggs, 2016 ▸). *TOMOMAN* has a module for exposure filtering on a per-tilt or per-frame basis using the empirical values determined by Grant & Grigorieff (2015 ▸). Frame-based exposure filtering keeps track of cumulative electron exposure and uses a modified normalization filter; frame-based filtering also requires aligned frame stacks, which can be generated by *MotionCor2*.

### 2.6. Tilt-series alignment

The next step in the preprocessing workflow is to determine the tilt-series alignment parameters. There are two approaches to performing tilt-series alignment: fiducial-based alignment where distinct objects such as gold nanoparticles are applied to the specimen and tracked as points across the tilt-series, or fiducial-less approaches that make direct use of the image data. *TOMOMAN* includes modules for performing tilt-series alignment using either *IMOD* (Mastronarde & Held, 2017 ▸) or *AreTomo* (Zheng *et al.*, 2022 ▸).

*TOMOMAN*’s *imod_preprocess* module uses the *IMOD etomo* package to perform initial tasks of coarse alignment and generation of necessary files for various steps within *IMOD* tilt-series alignment workflow. Users can adopt either the fiducial-based or the fiducial-less (patch tracking) *etomo* approach. After initial steps are performed, *IMOD*’s tilt-series alignment workflow requires manual curation steps, which are performed outside of *TOMOMAN* in the *etomo* graphical user interface.

For fully automated fiducial-less tilt-series alignment, a module for *AreTomo* is also included. *TOMOMAN* offers additional functionality on top of *AreTomo* by providing extra parameters, such as user-defined binning of tilt-series prior to alignment, the use of unfiltered or exposure-filtered tilt-series as inputs, and per-tomogram thickness values. We find that these additions can be helpful when tuning parameters for specific datasets. For example, in processing EMPIAR-11658 (see Section 4), we used exposure-filtered tilt-series that were binned 8× for alignment, as we found this enhanced signal-to-noise ratio visually improved the alignment quality and reduced computational cost. These parameters were set in the *TOMOMAN AreTomo* parameter file and *TOMOMAN* managed the correct tilt-series, Fourier binning via *IMOD* and appropriate rescaling of *AreTomo*’s outputs to the unbinned pixel size.

### 2.7. CTF estimation

As with single-particle images, high-resolution STA requires estimation and correction of the CTF. For CTF estimation, *TOMOMAN* includes modules for *CTFFIND4* (Rohou & Grigorieff, 2015 ▸), for which CTF estimation is independent of tilt-series alignment.

Additionally, *TOMOMAN* includes *tiltCTF*, an algorithm we developed that uses the determined tilt-series alignment parameters to generate power spectra that account for the tilt-dependent defocus gradient (Khavnekar & Wan, 2024 ▸). These power spectra are then supplied to *CTFFIND4* for Thon ring fitting.

### 2.8. Tomogram reconstruction

**Figure 3:**
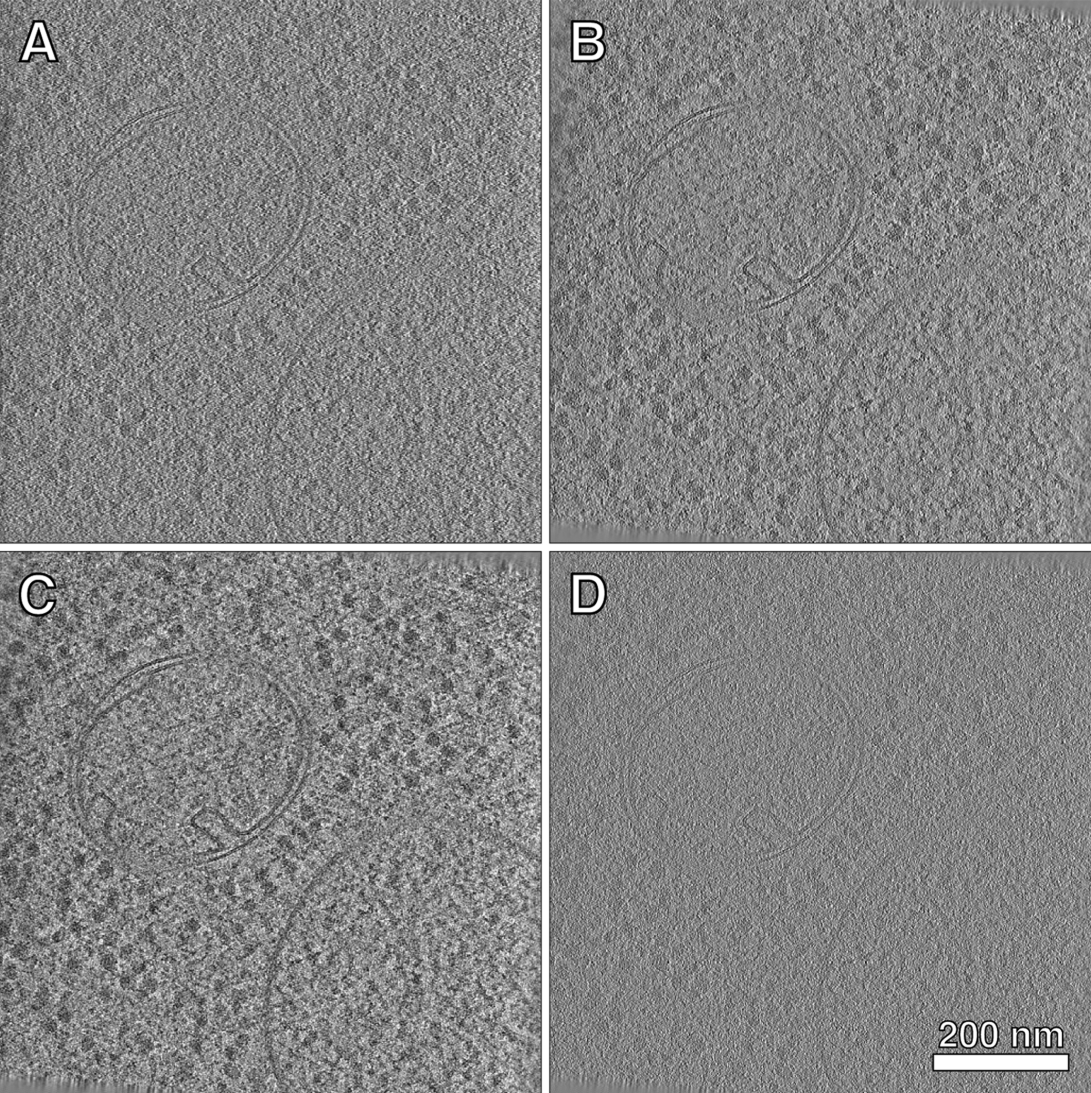
Comparison of tomograms reconstructed with different algorithms. (*a*) Non-CTF-corrected tomogram using WBP in *AreTomo*, which is reconstructed during tilt-series alignment, (*b*) denoised with *cryoCARE* (tomograms used for training and inference are reconstructed with WBP in *IMOD* using odd and even frames) and (*c*) reconstructed using 15 iterations of a SIRT-like filter in *IMOD*. (*d*) Tomogram reconstructed with 3D-CTF correction and WBP using *novaCTF*.

After tilt-series alignment, *TOMOMAN* can use the determined alignment parameters to reconstruct tomograms using different algorithms (Fig. 3 ▸), depending on the needs of the user. Tomograms reconstructed using weighted back projection (WBP) without CTF correction are typically output by the tilt-series alignment software [Fig. 3 ▸(*a*)]; these can be useful for quick visualization or low-resolution STA, but are otherwise limited in use.

*TOMOMAN* can also reconstruct contrast-enhanced tomograms, which can be useful for visual analysis [Figs. 3 ▸(*b*) and 3 ▸(*c*)]. For denoising using *noise2noise* methods such as *cryoCARE* [Fig. 3 ▸(*b*)] (Buchholz *et al.*, 2019 ▸), *TOMOMAN* can generate tomograms using odd and even sums of motion-corrected frames, which are necessary for these algorithms. Options for generating the required odd and even frame sums and corresponding tilt-series are included as parameters during the motion-correction task (see Section 2.3). Additionally, *TOMOMAN* can reconstruct tomograms in *IMOD* using WBP and the SIRT-like (simultaneous iterative reconstructive technique) filter [Fig. 3 ▸(*c*)]. After tilt-series alignment and CTF estimation, CTF-corrected tomograms can be reconstructed.

*TOMOMAN* also includes a module to generate 2D CTF-corrected tomograms using the *IMOD tilt* and *ctfphaseflip* programs (Xiong *et al.*, 2009 ▸) as well as 3D CTF-correction using *novaCTF* (Turoňová *et al.*, 2017 ▸) [Fig. 3 ▸(*d*)]. For *novaCTF*, *TOMOMAN* will generate the appropriate scripts as well as temporary and output directories for running *novaCTF*. After reconstruction, *TOMOMAN* will also perform any desired tomogram binning by Fourier cropping using the program *Fourier3D* (Turoňová, 2020 ▸). CTF-corrected tomograms can then be used for subsequent high-resolution STA.

### 2.9. Tomogram denoising

It is becoming a common practice to use machine-learning methods to enhance the contrast in tomograms through denoising. Contrast-enhanced or denoised tomograms allow for easier interpretation of molecular features by the human eye. *TOMOMAN* supports the generation of denoised tomograms using *cryoCARE* (Buchholz *et al.*, 2019 ▸). *TOMOMAN* handles generating tomograms from odd and even frames (see above), and handles the generation of necessary training files and the execution of *cryoCARE*.

### 2.10. Automated pipelines

Although all the above tasks can be run individually, it is often convenient to run as many tasks as possible in an automated workflow, particularly when preprocessing large high-throughput datasets. *TOMOMAN* allows users to define the various tasks of their preprocessing workflows into a ‘pipeline’ (*i.e.* a list of preprocessing tasks) which can then be run on high-performance computing clusters.

### 2.11. Interoperability with STA workflows

In addition to the core preprocessing workflow described in the previous section, *TOMOMAN* includes additional tools to export *TOMOMAN* metadata to other STA workflows. These include directly exporting to *STOPGAP* (Wan *et al.*, 2024 ▸), *Warp/RELION3/M* (Tegunov & Cramer, 2019 ▸; Bharat & Scheres, 2016 ▸; Tegunov *et al.*, 2021 ▸) and *RELION4* (Zivanov *et al.*, 2022 ▸) as well as moving between each of these workflows. For the *Warp/RELION/M* workflow, *TOMOMAN* also handles motion-corrected tilt images, while excluding those removed during curation, corresponding .mdoc files, tilt-series alignment files and the *RELION3*-formatted particle list. For the *RELION4* tomography workflow, *TOMOMAN* handles curated tilt-series, tilt-series alignment and CTF estimation parameters, additional files with order of tilt-series acquisition, and the per-tomogram particle list in *RELION4* tomography star file format. For *STOPGAP*, to generate required metadata files, such as wedge lists, functions are provided to export them directly from the tomolist. Additionally, *TOMOMAN* can convert particle metadata from the *STOPGAP* particle list into *RELION* star file format.

## 3. Archival and data sharing

### 3.1. Minimal projects for archiving

*TOMOMAN* includes an archival module to export the *TOMOMAN* project to a ‘minimal project’ that can be deposited in repositories such as EMPIAR (Iudin *et al.*, 2023 ▸). Minimal projects retain the *TOMOMAN* directory structure, the tomolist, and only the files necessary to revive the project at its current preprocessing state at a later time point or at another location, while cleaning up unnecessary intermediate files generated during preprocessing. The ability to revive a project in its exact state is key to reproducibility, as small changes in preprocessing can significantly affect downstream results. For example, slight differences in fiducial centering cause differences in tilt-series alignment and subsequent tomogram reconstruction; this affects the particle positions and orientations within tomograms as well as the resolution of STA structures. The *TOMOMAN* minimal project also minimizes the size of the project directory, as it is only necessary to keep the raw frames, the motion-corrected tilt stacks, and the metadata and parameter files, such as estimated CTF parameters and tilt-series alignment parameters. These 2D imaging data are typically only 2–3 Gb in size, whereas metadata and parameter files are on the order of kilobytes. These data are sufficient to reconstruct tomograms for subsequent STA workflows. This reduction in size makes it easier to share data, as large files such as tomograms, which can be hundreds of gigabytes, can often be reconstructed faster than they can be transmitted.

### 3.2. Community data sharing and distributed collaborative cryo-ET

*TOMOMAN* minimal projects allow users to effectively restore projects to their exact previous preprocessing states. In addition to being key to reproducibility and archiving, this enables distribution of preprocessed projects between collaborators for more complex downstream processing such as STA. This enables large-scale cellular cryo-ET projects, where different laboratories can focus on their specific molecules or subcellular structures of interest.

## 4. Conclusions

A major challenge in cryo-ET and STA is how to leverage the unique capabilities of the wide variety of packages available for each step of the image-processing workflow. This is typically due to the difficulty in managing the metadata between these packages, which typically have different file and parameter formats. *TOMOMAN* addresses these issues by establishing its own internal metadata format and providing extensible modules to interface with other software packages, allowing users to develop their own package-agnostic workflows that are best suited to their biological problems.

*TOMOMAN*, an open-source package written in MATLAB, is supplied as a standalone package that can be executed using the freely available MATLAB runtime. *TOMOMAN* is designed with high-performance computing in mind; it generates all the necessary scripts to launch external packages with defined computational resources, allowing users to seamlessly run custom workflows in parallel. Beyond the initial cryo-ET preprocessing tasks, the *TOMOMAN* metadata tracking enables the transfer of projects between different STA workflows including *Warp/RELION3/M*, *RELION4* and *STOPGAP*.

Other cryo-ET packages such as *IMOD* (Kremer *et al.*, 1996 ▸), *Warp* (Tegunov & Cramer, 2019 ▸), *EMAN2* (Tang *et al.*, 2007 ▸; Chen *et al.*, 2019 ▸), *ScipionTomo* (Jiménez de la Morena *et al.*, 2022 ▸), *TomoBEAR* (Balyschew *et al.*, 2023 ▸) and *RELION5* (Burt *et al.*, 2024 ▸) also offer start-to-end workflows for tilt-series preprocessing. However, these packages generally aim to offer a simplified streamlined workflow that may not be suitable for each biological problem. *IMOD*, *Warp* and *EMAN2* primarily use their own internal functions for preprocessing tasks and tomographic reconstruction, while *RELION5* now includes a cryo-ET workflow that provides wrapper scripts for external packages to perform CTF estimation, tilt-series alignment and denoising and an internal tomogram reconstruction algorithm. *ScipionTomo*, on the other hand, offers a workflow manager where individual packages can be added as plugins. *ScipionTomo* integration occurs at a relatively low level within the package; this process can be time consuming as it requires expertise in *ScipionTomo* and the package to be integrated. This is distinct from *TOMOMAN*, which does not directly integrate packages, but instead generates scripts for running external packages and functions for capturing the outputs and storing the necessary metadata in its internal format. *TomoBEAR* is similar in functionality to *TOMOMAN* in that it wraps external packages and runs scripted workflows, but it is aimed at minimizing intermediate steps to deliver a streamlined linear STA workflow. *TOMOMAN* also streamlines the interoperability of various packages, but aims to facilitate the testing and use of different packages for each preprocessing step. This flexibility allows users to optimize workflows to their specific biological problems.

One unique feature of *TOMOMAN* is its archival functions, which streamline data deposition to community databases such as EMPIAR while also providing the necessary metadata for users to download datasets and restart projects. This enables the sharing of information-rich cryo-ET datasets without the need for downstream users to reprocess data, thereby reducing overall computational costs and ensuring reproducibility between laboratories. This aspect of reproducibility is particularly important, as subtle changes in preprocessing can significantly affect downstream results. We believe these archival functions will help enable large-scale consortium cryo-ET projects, while also opening the data to the wider biological community. *TOMOMAN* has already been used to manage a number of projects, including some that have been deposited in EMPIAR as *TOMOMAN* minimal projects such as EMPIAR-11830 (Khavnekar *et al.*, 2023*a* ▸), EMPIAR-11756 (Khavnekar *et al.*, 2023*a* ▸), EMPIAR-11658 (Wan *et al.*, 2024 ▸; Rangan *et al.*, 2023 ▸), EMPIAR-11398 (Khavnekar *et al.*, 2022 ▸), EMPIAR-11325, EMPIAR-11324 and EMPIAR-11322 (Khavnekar *et al.*, 2023*b* ▸). In particular, the EMPIAR-11830 project was also processed as a multi-institutional multi-user collaborative *TOMOMAN* project. We envision that these *TOMOMAN* minimal project depositions together with well annotated metadata will be important for the development of large data approaches, such as novel AI-based image-processing tools.

Altogether, *TOMOMAN* offers a solution for developing comprehensive cryo-ET workflows that are accessible for both experienced and novice users. The software package is available at https://github.com/wan-lab-vanderbilt/TOMOMAN.
